# GBP1-CDK9-STAT3 signaling axis promotes osteosarcoma PD-L1 expression and immune escape

**DOI:** 10.1016/j.neo.2025.101232

**Published:** 2025-09-20

**Authors:** Doudou Jing, Binghong Chen, Ruqi Liang, Fei Li, Bin Zhao, Feifei Pu, Wei Wu

**Affiliations:** a Department of Orthopaedics, The Second Hospital of Shanxi Medical University, Taiyuan, 030001, China; b College of Chemistry and Chemical Engineering, Taiyuan University of Technology, Taiyuan 030002, China; c Department of Orthopaedics, Traditional Chinese and Western Medicine Hospital of Wuhan, Tongji Medical College, Huazhong University of Science and Technology, Wuhan 430022, China; d Department of Orthopaedics, Wuhan No.1 Hospital, Wuhan 430022, China; e Department of Orthopedics, Union Hospital, Tongji Medical College, Huazhong University of Science and Technology, Wuhan, China

**Keywords:** Osteosarcoma, Immune escape, Cancer immunotherapy, PD-L1

## Abstract

Osteosarcoma is a common malignant bone tumor, characterized by its high invasiveness and propensity for lung metastasis. Despite advances in treatment, clinical outcomes remain poor, and patient prognosis is still unsatisfactory. Therefore, the development of more effective therapies is urgently needed. Here, we demonstrate that differential expression of GBP1 significantly influences PD-L1 expression and mediates immune escape in osteosarcoma. Specifically, our results reveal that GBP1 regulates PD-L1 expression by activating CDK9 and promoting STAT3 phosphorylation. These findings suggest that targeting GBP1 may represent a promising therapeutic strategy for the treatment of osteosarcoma by impairing tumor immune evasion.

## Introduction

Osteosarcoma (OS) is the most prevalent primary bone malignancy, marked by high local invasiveness and propensity for lung metastasis. Despite progress in neoadjuvant chemotherapy and surgical interventions, the five-year survival rate for OS patients remains approximately 70 %. However, for patients exhibiting poor chemotherapy response, survival rates drop to around 30 % [1,2], highlighting the urgent need for improved therapeutic strategies for osteosarcoma. Recently, immune checkpoint inhibitors (ICIs), targeting cytotoxic T lymphocyte-associated protein 4 (CTLA-4), programmed cell death protein 1 (PD-1), and its ligand programmed death-ligand 1 (PD-L1), have significantly advanced cancer therapy, improving survival outcomes for malignancies such as melanoma, non-small cell lung cancer, and renal cell carcinoma [3]. Although osteosarcoma, characterized by a highly unstable genome, elevated tumor mutational burden, and active immune infiltration, theoretically presents a suitable candidate for immunotherapy [4], clinical studies have shown limited ICI efficacy in osteosarcoma, with response rates remaining around 10–20 % [[5], [6], [7], [8]].

Guanylate-binding protein 1 (GBP1), an interferon-stimulated gene induced by interferon-gamma (IFN-γ), has been implicated in tumor progression and treatment resistance in various cancers, including ovarian and head and neck cancers [9]. GBP1 has been shown to promote proliferation, migration, and metastasis in lung cancer by binding to indoleamine 2,3-dioxygenase 1 (IDO1) and to enhance resistance to epidermal growth factor receptor (EGFR) inhibitors by interacting with phosphoglycerate kinase 1 (PGK1) [10,11]. In this study, we observed a positive correlation between GBP1 and PD-L1 expression at both mRNA and protein levels in osteosarcoma tissues. Using unbiased mass spectrometry following immunoprecipitation with GBP1 antibodies, we identified STAT3 as a potential mediator of GBP1-induced PD-L1 upregulation. Further experiments revealed that cyclin-dependent kinase 9 (CDK9) may bind to GBP1, pwhich promotes STAT3 phosphorylation and nuclear translocation. These findings elucidate a novel GBP1-CDK9-STAT3 signaling axis that promotes PD-L1 expression and contributes to immune evasion in osteosarcoma, providing insights into potential therapeutic targets for enhancing immunotherapy efficacy in this malignancy.

## Materials and methods

### Cell culture

MNNG/HOS and U-2 OS cell lines were obtained from Wuhan Pricella Biotechnology Co., Ltd. and authenticated by short tandem repeat (STR) analysis. MNNG/HOS cells were cultured in DMEM with 10 % fetal bovine serum (FBS) and 1 % penicillin-streptomycin (P/S) (Thermo Fisher Scientific, Waltham, MA, USA). U-2 OS cells were cultured in McCoy's 5A medium with 10 % FBS and 1 % P/S (Thermo Fisher Scientific, USA). All cultures were maintained in a 5 % CO₂ humidified incubator at 37°C.

### Chemical agents, siRNAs, and plasmids

The STAT3 inhibitor STAT3-IN-13 (HY-150603) and the CDK9 inhibitor LDC000067 (Synonyms: LDC067, HY-15878) were purchased from MedChemExpress (Monmouth Junction, USA). siRNAs were procured from RiboBio (Guangzhou, China). The GBP1 cDNA was cloned into the pCDNA3.1-FLAG-C vector, while the STAT3 cDNA was inserted into the pCMV-6 × His-Neo vector. Specific shRNAs were synthesized and cloned into the pLV3-U6-shRNA1-CopGFP-Puro vector. The sequences of all constructs are listed in Table S1.

### Quantitative RT-PCR

Total RNA was isolated using Trizol reagent (Thermo Fisher Scientific, USA). One microgram of RNA was reverse-transcribed using the PrimeScript™ RT kit (Takara, Japan). Quantitative real-time PCR was conducted with the TB Green Premix® Ex Taq™ kit (Takara, Japan) following the manufacturer’s protocol. β-actin served as the internal control. Primer sequences are provided in Table S2.

### Colony formation and transwell assay

Cells were seeded into 6-well plates at specified densities and transfected with appropriate constructs. After incubation for the designated period, cells were fixed and stained with 0.5 % crystal violet solution. Stained cells were imaged and quantified using ImageJ software.

### Immunohistochemistry (IHC) and clinical specimen collection

Osteosarcoma tissue microarray slides (#L072Bn01) were purchased from Zhongke Guanghua (Xi’an) Intelligent Biotechnology Co., Ltd. Standard immunohistochemical procedures were performed, and tissue-specific antibodies were used for staining. The antibodies included GBP1 (#15303-1-AP, Proteintech, 1:250) and PD-L1 (#66248-1-Ig, Proteintech, 1:3000). Staining intensity (SI) was calculated as follows: SI = (percentage of positive cells × staining intensity).

### In vivo research

All animal experiments were approved by the Experimental Animal Ethics Committee of the Second Hospital of Shanxi Medical University. Six-week-old BALB/C-nude mice were obtained from SJA Laboratory Animal Company (Changsha, China) and were used in the experiments. At 48 hours post-transfection, experimental cells (5 × 10⁶ cells/mouse) were subcutaneously injected into the left flanks of each mouse. Tumor dimensions were measured with calipers, and tumor volume was calculated as (L × W²)/2. Tumors were excised, measured, and weighed post-euthanasia.

### Statistical analysis

All data are expressed as mean ± standard deviation (SD). For single comparisons, a paired Student's t-test was used, while one-way ANOVA with post-hoc testing was performed for multiple comparisons. Statistical significance was set at *, P < 0.05, **, P < 0.01, or ***, P < 0.001. Results are presented as mean ± SD (n=3).

## Results

### High expression of PD-L1 may be associated with elevated GBP1 expression

**Fig. 1:**
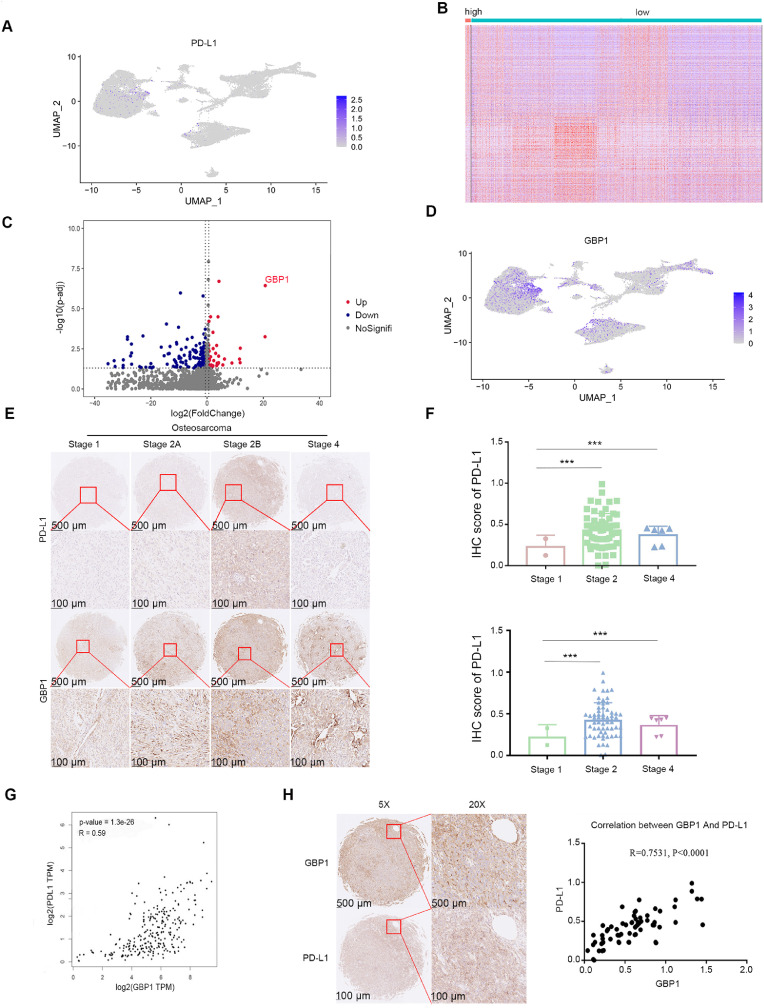
GBP1 is upregulated and positively correlated with PD-L1 expression in osteosarcoma.

To investigate the molecular mechanisms underlying elevated PD-L1 expression in osteosarcoma, we analyzed single-cell sequencing data from osteosarcoma cells (GSE162454). This analysis identified 353 genes that were differentially expressed between PD-L1-positive and PD-L1-negative tumor cell populations, with 111 genes upregulated and 242 downregulated (Fig. 1A-C). Notably, Guanylate-Binding Protein 1 (GBP1) mRNA levels were significantly higher in PD-L1-positive tumor cells (Fig. 1D). Immunohistochemistry (IHC) analysis using a PD-L1-specific antibody on osteosarcoma tissue microarrays showed significantly increased levels of PD-L1 expression in stage 2 osteosarcoma tissues. Additionally, IHC staining with a GBP1-specific antibody confirmed the upregulation of GBP1 in stage 2 osteosarcoma tissues, in parallel with PD-L1 expression (Fig. 1E, F). These observations were consistent with an analysis of the Cancer Genome Atlas (TCGA) Sarcoma (SARC) dataset, which revealed significantly higher PD-L1 mRNA expression in sarcoma tissues compared to normal tissues (Fig. S1A).

A: Single-cell RNA sequencing (GSE162454) showing PD-L1 expression across osteosarcoma tumor cell populations. B, C: Differential gene expression analysis between PD-L1-positive and PD-L1-negative tumor cells, presented as heatmap (B) and volcano plot (C). D: Single-cell RNA sequencing analysis showing elevated GBP1 expression in PD-L1-positive tumor cells. E, F: Immunohistochemistry (IHC) of osteosarcoma tissue microarrays using PD-L1 and GBP1 antibodies, showing representative images (E) and quantified expression levels across tumor stages (F). ***, P < 0.001. G: Correlation analysis between GBP1 and PD-L1 expression in sarcoma tissues versus normal tissues using the GEPIA platform. H: Representative IHC images and correlation analysis of GBP1 and PD-L1 in osteosarcoma tissue microarrays.

Using the GEPIA platform, we identified a positive correlation between GBP1 and PD-L1 expression in sarcoma tissues (Fig. 1G), a finding further supported by IHC analysis in osteosarcoma tissues (Fig. 1H).

### GBP1 enhances proliferation, migration, and invasion of osteosarcoma cells

**Fig. 2:**
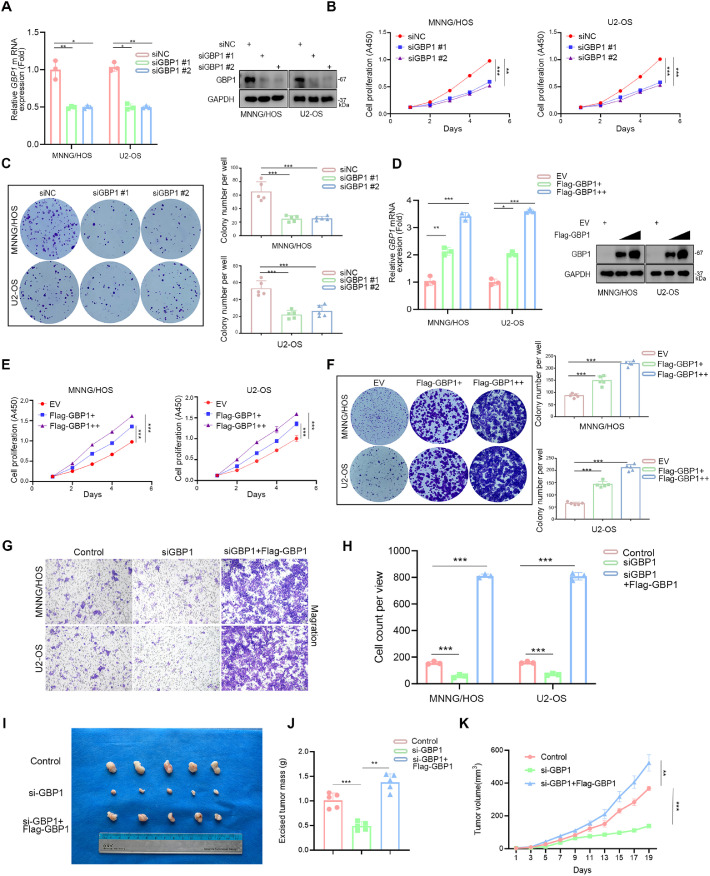
GBP1 promotes cell proliferation, migration, and invasion in osteosarcoma.

GBP1 knockdown by siRNA markedly reduced its expression in osteosarcoma cells (Fig. 2A). Silencing GBP1 significantly suppressed cell proliferation, as shown by CCK-8 and colony formation assays in both MNNG/HOS and U2-OS cells (Fig. 2B, C). In contrast, FLAG-GBP1 overexpression significantly increased osteosarcoma cell proliferation, demonstrated by CCK-8 and colony formation analyses. (Fig. 2D-F). Transwell assays further revealed that GBP1 silencing impaired, whereas GBP1 overexpression promoted, migration and invasion of osteosarcoma cells (Fig. 2G, H; Fig. S1D, E). Consistently, in vivo xenograft experiments demonstrated that GBP1 depletion suppressed tumor growth, while GBP1 re-expression rescued tumorigenic potential (Fig. 2I-K).

A-C: MNNG/HOS and U2-OS cells were transfected with the indicated siRNAs for 48 hours. GBP1 expression was assessed by RT-qPCR and Western blotting (A). Cell proliferation was evaluated using CCK-8 (B) and colony formation assays (C). D-F: MNNG/HOS and U2-OS cells were transfected with the indicated plasmids for 48 hours. GBP1 overexpression was confirmed by RT-qPCR and Western blotting (D). Proliferation was assessed by CCK-8 (E) and colony formation assays (F). G, H: Cells were transfected with siRNAs or plasmids for 48 hours. Transwell assays were performed to evaluate migration and invasion.I-K: Cells were subcutaneously injected into nude mice. Representative tumor images (I), tumor weights (J), and growth curves (K) are shown. All data are presented as mean ± SD. Experiments were performed in triplicate (A, D, G, H) or quintuplicate (B, C, E, F, I – K). *, P < 0.05; **, P < 0.01; ***, P < 0.001.

### GBP1 promotes PD-L1 expression through interaction with STAT3

**Fig. 3:**
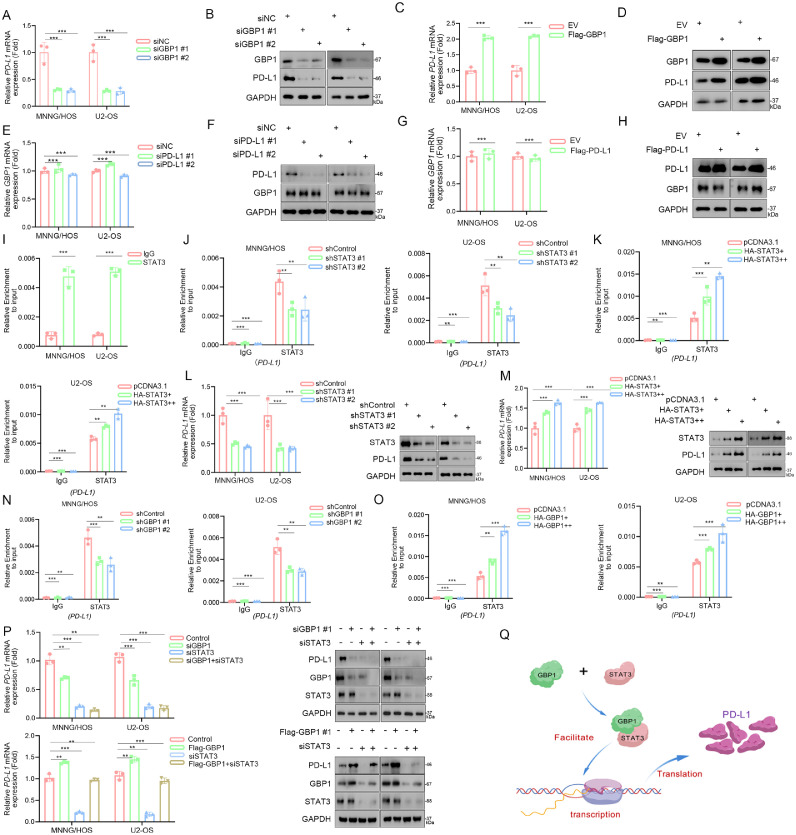
GBP1 promotes PD-L1 expression through interaction with STAT3.

To elucidate whether GBP1 modulates PD-L1 expression, we silenced GBP1 in MNNG/HOS and U2-OS cells and found that PD-L1 protein and mRNA levels were significantly reduced (Fig. 3A, 3B). Conversely, GBP1 overexpression markedly increased PD-L1 expression (Fig. 3C, 3D). To test whether PD-L1 regulates GBP1, we modulated PD-L1 expression and observed no changes in GBP1 protein or mRNA levels, suggesting a unidirectional regulation from GBP1 to PD-L1 (Fig. 3E-H).

A-H: Knockdown of GBP1 decreased, whereas its overexpression increased, PD-L1 mRNA and protein levels. In contrast, PD-L1 knockdown or overexpression did not affect GBP1 levels, as assessed by RT-qPCR and Western blotting. I: ChIP-qPCR confirmed STAT3 binding to the PD-L1 promoter. J-M: STAT3 knockdown reduced, whereas STAT3 overexpression enhanced, both STAT3 binding to the PD-L1 promoter and PD-L1 expression. N–O: GBP1 knockdown reduced, while GBP1 overexpression increased STAT3 binding to the PD-L1 promoter. P: Rescue experiments showed that GBP1 regulates PD-L1 expression in a STAT3-dependent manner. Q: Schematic model illustrating that GBP1 promotes STAT3 recruitment to the PD-L1 promoter, thereby upregulating PD-L1 transcription and translation. All data are mean ± SD of three independent experiments. *, P < 0.05; **, P < 0.01; ***, P < 0.001.

To explore the underlying mechanism, we performed Co-immunoprecipitation mass spectrometry (CoIP-MS) and identified STAT3 as a GBP1-interacting protein. STAT3 belongs to the signal transducer and activator of transcription family and plays a pivotal role in mediating cytokine and growth factor signaling. Under physiological conditions, STAT3 activity is tightly regulated; however, in many tumors, STAT3 is constitutively activated and has been shown to drive malignant progression. Notably, previous studies reported that STAT3 promotes PD-L1 transcription, linking it to tumor immune evasion [12,13].

Consistent with this, ChIP-qPCR confirmed STAT3 binding to the PD-L1 promoter (Fig. 3I). This binding was reduced by STAT3 knockdown and enhanced by STAT3 overexpression (Fig. 3J, K). Accordingly, STAT3 silencing suppressed, whereas STAT3 overexpression increased, PD-L1 expression (Fig. 3L, M). Importantly, GBP1 knockdown reduced, while GBP1 overexpression enhanced STAT3 occupancy at the PD-L1 promoter (Fig. 3N, O). Rescue experiments further demonstrated that the effects of GBP1 on PD-L1 expression are dependent on STAT3 activity (Fig. 3P). Together, these findings indicate that GBP1 upregulates PD-L1 expression by facilitating STAT3 binding to its promoter.

### GBP1 interacts with STAT3 to promote its phosphorylation and PD-L1 expression

**Fig. 4:**
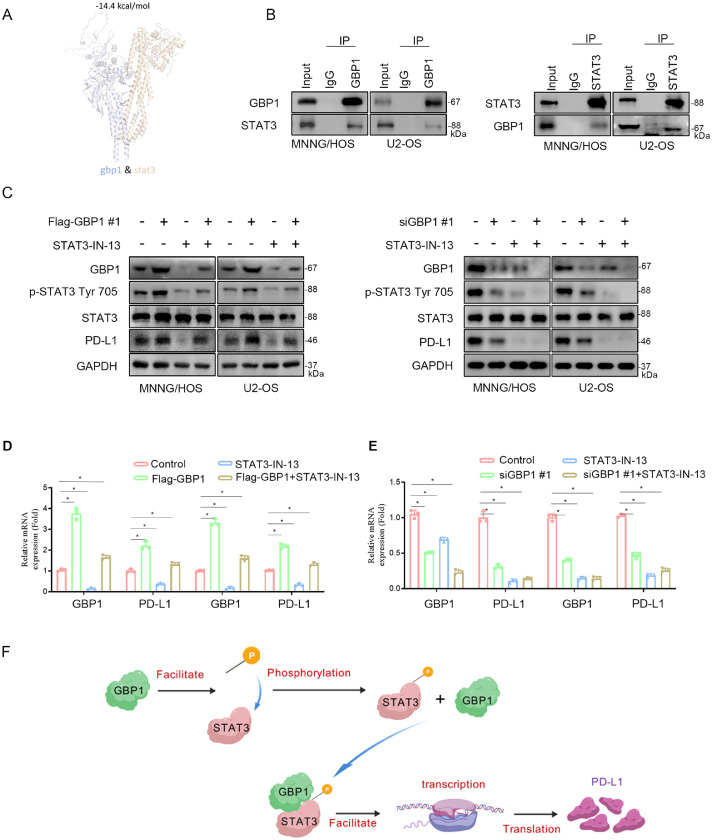
GBP1 promotes STAT3 Tyr705 phosphorylation.

To verify the interaction between STAT3 and GBP1, protein docking analysis revealed multiple hydrogen bonds between GBP1 and STAT3 (Fig. 4A). Co-immunoprecipitation further confirmed their physical association in both MNNG/HOS and U2-OS cells (Fig. 4B). To determine an appropriate concentration of the STAT3 inhibitor for functional assays, we measured the IC_50_ values of STAT3-IN-13 for inhibiting STAT3 Tyr705 phosphorylation, which were 0.1694 μM in MNNG/HOS and 0.1986 μM in U2-OS (Fig. S1B). Based on these results, we selected 0.15 μM for subsequent rescue experiments. At this concentration, overexpression of GBP1 increased STAT3 Tyr705 phosphorylation and PD-L1 expression, whereas GBP1 knockdown produced the opposite effects. Importantly, treatment with STAT3-IN-13 abrogated GBP1-induced STAT3 activation and PD-L1 upregulation at both the protein (Fig. 4C) and mRNA levels (Fig. 4D, E).

A: Structure of the STAT3-GBP1 complex displayed using PyMOL. B: Western blot analysis after co-immunoprecipitation in MNNG/HOS and U2-OS cell lines using STAT3 or GBP1 antibodies. C-E: Western blot and RT-qPCR analyses showing that GBP1 overexpression enhanced, while GBP1 knockdown reduced, STAT3 Tyr705 phosphorylation and PD-L1 expression. Treatment with 0.15 µM STAT3-IN-13 abrogated these effects. Data are presented as mean ± SD of three independent experiments. *, P < 0.05; ***, P < 0.001. F: Schematic model indicating that GBP1 phosphorylates STAT3-T705 to regulate PD-L1 expression.

### GBP1 promotes STAT3 phosphorylation through interaction with CDK9

**Fig. 5:**
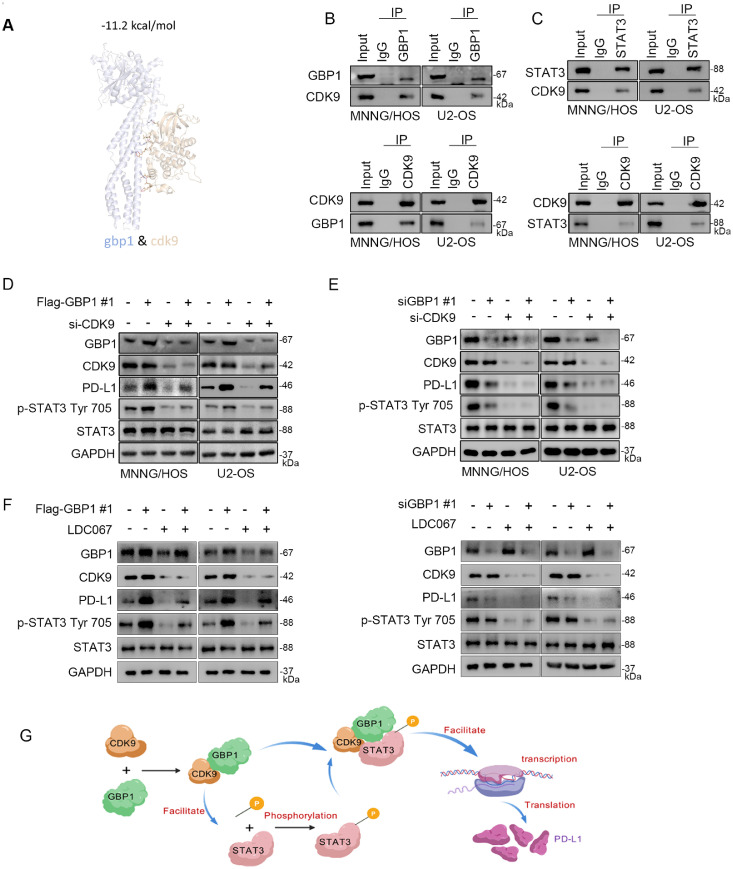
GBP1 drives STAT3 activation and PD-L1 upregulation through CDK9.

CDK9, a serine/threonine protein kinase of the cyclin-dependent kinase family, regulates transcription by phosphorylating RNA polymerase II and has been implicated in oncogenesis and tumor immune evasion [[14], [15], [16]]. Protein docking analyses revealed multiple hydrogen bonds between GBP1 and CDK9 (Fig. 5A). Co-immunoprecipitation confirmed that CDK9 interacts with both GBP1 and STAT3 in osteosarcoma cells (Fig. 5B, C).

A: Predicted docking model of the GBP1–CDK9 complex. B, C: Western blot analysis after co-immunoprecipitation in MNNG/HOS and U2-OS cell lines using GBP1, CDK9, or STAT3 antibodies. D-F: Western blot analyses showing that CDK9 knockdown reduced STAT3 Tyr705 phosphorylation and PD-L1 expression, which were partially rescued by GBP1 overexpression. Combined knockdown of GBP1 and CDK9 synergistically suppressed STAT3 phosphorylation and PD-L1 expression. Treatment with the CDK9 inhibitor LDC067 (5 µM, 24 h) also inhibited STAT3 phosphorylation and PD-L1 expression, which was partially rescued by GBP1 overexpression, while GBP1 knockdown together with LDC067 treatment further enhanced the suppression. G: Schematic model of the GBP1–CDK9–STAT3 signaling axis regulating PD-L1 expression in osteosarcoma.

Knockdown of CDK9 markedly reduced STAT3 Tyr705 phosphorylation and PD-L1 expression, whereas GBP1 overexpression partially rescued these effects (Fig. 5D). Combined knockdown of GBP1 and CDK9 synergistically suppressed STAT3 phosphorylation and PD-L1 expression (Fig. 5E). To further validate the role of CDK9, we determined the cellular IC_50_ values of the CDK9 inhibitor LDC067, which were 8.394 μM in MNNG/HOS and 6.104 μM in U2-OS (Fig. S1C). Consistent with the genetic data, treatment with LDC067 significantly inhibited GBP1-induced STAT3 phosphorylation and PD-L1 expression (Fig. 5F). Collectively, these findings suggest that GBP1 promotes STAT3 activation through interaction with CDK9, thereby facilitating PD-L1 expression.

## Discussion

Guanylate-binding protein 1 (GBP1) is an interferon-γ-induced GTPase [17]. Interferon-γ (IFN-γ) acts as a central regulator of immune responses, primarily signaling through the Janus kinase (JAK) and Signal Transducer and Activator of Transcription (STAT) pathways [18]. IFN-γ has been recognized as a key factor influencing the efficacy of immunotherapy [19]. Prior studies have demonstrated that GBP1 interaction with SP1 promotes STAT3 activation, which enhances proliferation and invasion in cutaneous squamous cell carcinoma [20]. In this study, we found that GBP1 influences the tumor immune environment of osteosarcoma by modulating PD-L1 transcription and translation.

STAT3, a member of the STAT family, facilitates signal transduction from extracellular cytokines and growth factors to the nucleus, initiating gene transcription. Under normal physiological conditions, STAT3 signaling is tightly regulated. However, in many tumors, STAT3 is constitutively activated, closely associated with tumor malignancy [21,22]. Post-translational modifications, including phosphorylation, regulate STAT3 activity by promoting its dimerization, nuclear translocation, and DNA-binding capacity. Specifically, Tyr705 phosphorylation plays a critical role in maintaining STAT3′s transcriptional activity [23].

To further elucidate the molecular mechanism by which GBP1 promotes STAT3 Tyr705 phosphorylation, we analyzed our mass spectrometry data, identifying cyclin-dependent kinase 9 (CDK9) as a potential GBP1-binding partner that could facilitate STAT3 phosphorylation and nuclear translocation. CDK9, a member of the serine/threonine kinase family, exists in two isoforms, CDK9-42 and CDK9-55, with CDK9-42 being more widely distributed. CDK9 typically forms a heterodimer with cyclin T1, T2a, or T2b, collectively known as the positive transcription elongation factor (p-TEFb). This complex regulates transcription by phosphorylating RNA polymerase II, influencing its pausing and initiation [14]. Dysregulated CDK9 activity is observed in various cancers, where it promotes cancer progression by regulating RNA transcription, DNA repair, recruitment of transcription factors, and tumor immune evasion [15,16]. Inhibition of CDK9 has shown synergy with other CDK inhibitors in enhancing anticancer activity [24]. Moreover, evidence suggests that CDK9 is associated with the phosphorylation status of STAT3 [25]. Notably, aberrant STAT3 activation has been observed in osteosarcoma patients and is linked to poor prognosis [26]. Here, we demonstrated that GBP1 increases STAT3 phosphorylation by binding to CDK9, which promotes STAT3 nuclear translocation and regulates PD-L1 transcription and translation.

In summary, our study reveals that GBP1 directly interacts with CDK9, leading to increased STAT3 Tyr705 phosphorylation. This, in turn, promotes STAT3 activation and nuclear translocation. Once activated, STAT3 binds to the promoters of PD-L1, enhancing their transcription. This signaling axis drives PD-L1 expression, facilitating immune evasion in osteosarcoma. These findings offer a detailed understanding of the molecular mechanism by which GBP1 contributes to PD-L1 upregulation in osteosarcoma and provide potential therapeutic targets for PD-L1-based immunotherapy.

## Availability of data and materials

The datasets generated during this study can be obtained by contacting the corresponding author with a reasonable request.

## Funding

This study was supported by The 10.13039/501100001809National Natural Science Foundation of China (grant no 82303192).

## Patient consent for publication

Not applicable.

## CRediT authorship contribution statement

**Doudou Jing:** Writing – original draft, Funding acquisition, Conceptualization. **Binghong Chen:** Writing – original draft, Data curation, Conceptualization. **Ruqi Liang:** Writing – original draft, Investigation, Formal analysis. **Fei Li:** Writing – original draft, Resources, Project administration. **Bin Zhao:** Writing – original draft, Visualization, Supervision. **Feifei Pu:** Writing – review & editing, Visualization, Supervision. **Wei Wu:** Writing – review & editing, Supervision.

## Declaration of competing interest

The authors declare that they have no known competing financial interests or personal relationships that could have appeared to influence the work reported in this paper.

## Appendix Supplementary materials

Image, image 1
