# Corrigendum to “GBP1-CDK9-STAT3 signaling axis promotes osteosarcoma PD-L1 expression and immune escape” [Neoplasia 69 (2025) 101232]

**DOI:** 10.1016/j.neo.2026.101273

**Published:** 2026-01-21

**Authors:** Doudou Jing, Binghong Chen, Ruqi Liang, Fei Li, Bin Zhao, Feifei Pu, Wei Wu

**Affiliations:** a Department of Orthopaedics, The Second Hospital of Shanxi Medical University, Taiyuan, 030001, China; b College of Chemistry and Chemical Engineering, Taiyuan University of Technology, Taiyuan, 030002, China; c Department of Orthopaedics, Traditional Chinese and Western Medicine Hospital of Wuhan, Tongji Medical College, Huazhong University of Science and Technology, Wuhan, 430022, China; d Department of Orthopaedics, Wuhan No.1 Hospital, Wuhan 430022, China; e Department of Orthopedics, Union Hospital, Tongji Medical College, Huazhong University of Science and Technology, Wuhan, 430015, China

The authors regret that in the original version of this article, Bin Zhao was not designated as a corresponding author. The authors wish to correct the author information to identify Bin Zhao as a co-corresponding author alongside Wei Wu and Feifei Pu. This change does not affect the scientific results or conclusions of the paper.

The authors would like to apologise for any inconvenience caused.

