# Supplementary figures and images for: GBP1-CDK9-STAT3 signaling axis promotes osteosarcoma PD-L1 expression and immune escape
Source: Neoplasia. 2025 Sep 20;69:101232. doi: 10.1016/j.neo.2025.101232 (PMC12489786; doi:10.1016/j.neo.2025.101232)

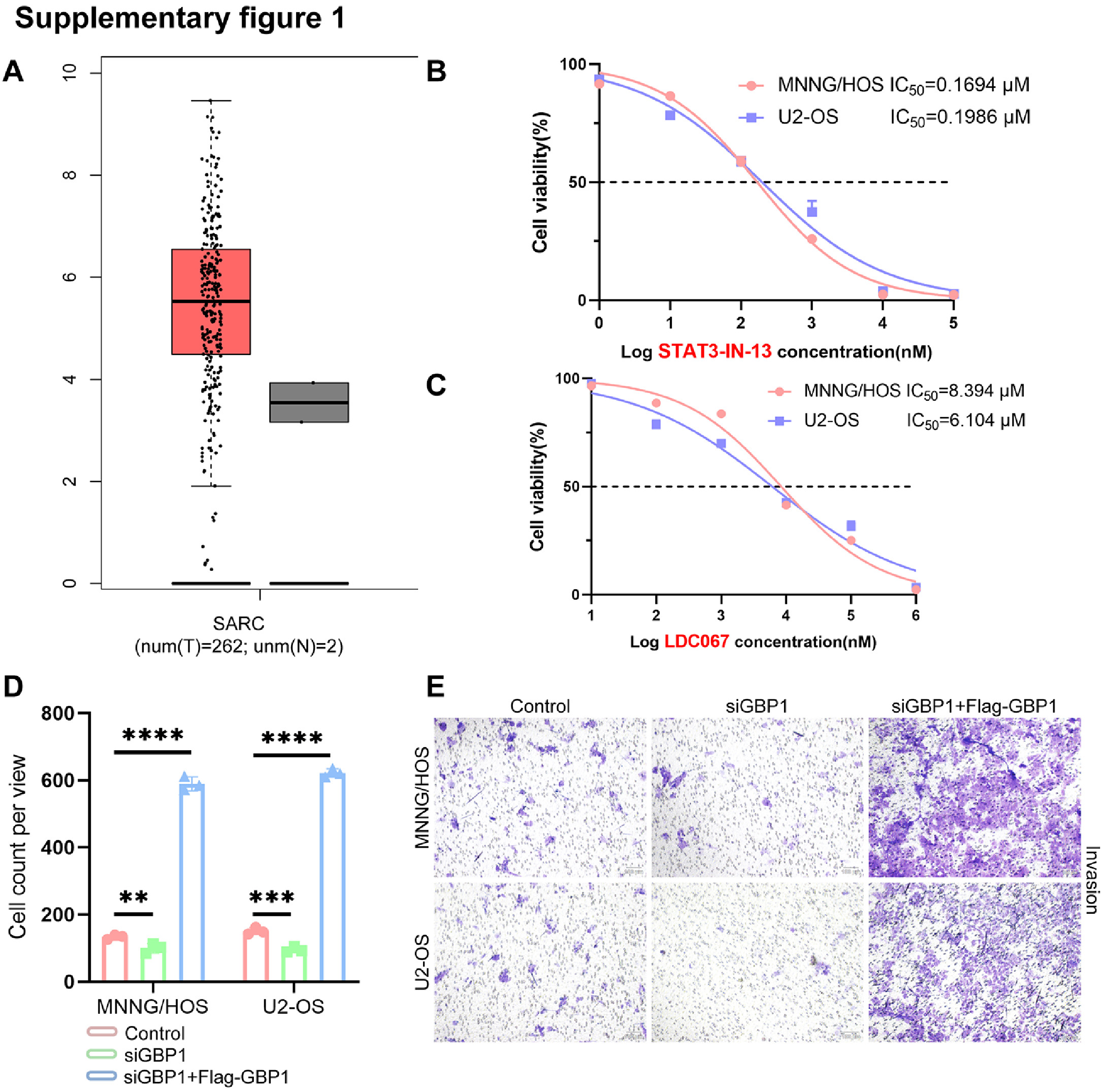

Supplement: Supplementary file 1 [file mmc1.jpg]
